# Nanoengineered polyglutamic acid fertilizers via self-assembly for efficient tomato growth

**DOI:** 10.3389/fpls.2025.1702462

**Published:** 2026-01-15

**Authors:** Jiangtao Dong, Hexin Li, Bowen Yuan, Donghui Zhang, Hongliang Wang, Tao Wang, Songwei Li, Runqiang Liu

**Affiliations:** 1 Henan Engineering Research Center of Green Pesticide Creation & Intelligent Pesticide Residue Sensor Detection, Henan Institute of Science and Technology, Xinxiang, Henan, China; 2 School of Plant Protection and Environment, Henan Institute of Science and Technology, Xinxiang, Henan, China; 3 School of Chemistry and Chemical Engineering, Henan Institute of Science and Technology, Xinxiang, Henan, China

**Keywords:** γ-polyglutamic acid, nanofertilizer, foliar absorption, bidirectional translocation, promote tomato growth, stress tolerance

## Abstract

**Introduction:**

Polyglutamic acid (γ-PGA) is a promising biostimulant for enhancing crop growth and stress resistance, while its agricultural application is limited by poor leaf retention, low mobility within plants, and susceptibility to rain wash-off.

**Methods:**

This study developed PGA nanofertilizers via a facile one-step self-assembly strategy in crude γ-PGA aqueous solution without adding salt ions to overcome these limitations. SEM images show that the obtained nanoparticles appear uniform spherical morphology and good dispersibility in water with an average hydrodynamic diameter of 182 nm confirmed by DLS. XRD and DSC patterns indicate a strong reduction in crystallinity consistent with a largely amorphous or highly disordered state.

**Results:**

Fluorescence imaging of FITC-labeled PGA nanofertilizers (FITC@PGA) indicates systemic, vascular-localized signals consistent with bidirectional movement from absorption of both roots and leaves. Importantly, the PGA nanofertilizers exhibited superior rainfastness and leaf retention compared to crude γ-PGA. Physiological assessments showed that foliar application of PGA nanofertilizers significantly enhanced chlorophyll content, root development, and antioxidant enzyme activities compared to that of crude γ-PGA, which led to significant improvement for tomato growth and stress tolerance.

**Discussion:**

It is clear shown that the nano-engineering strategy will provide a promising approach for developing efficient and eco-friendly nanofertilizer.

## Graphical Abstract

**Figure:**
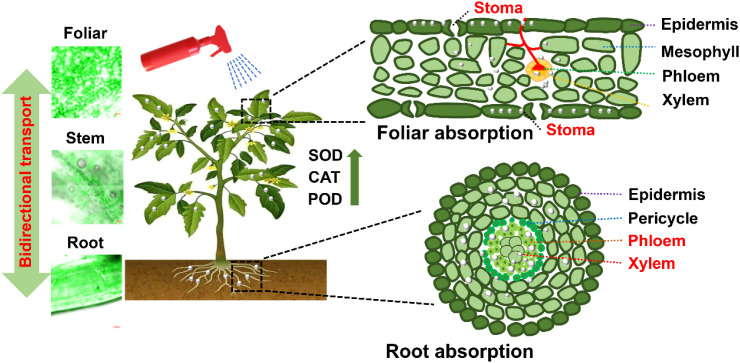
TOC graphics.

## 1 Introduction

Biostimulants are recognized as significant tools that enhance plant nutrient uptake, stimulate endogenous hormone secretion, and improve stress resistance (El Sherif et al., 2023; Liatile et al., 2022; Khalil et al., 2025). Their primary mechanism involves activating signal transduction pathways within plant cells to promote growth and development. Additionally, biostimulants bolster plant resistance against diseases and pests while strengthening adaptability to adverse environmental conditions such as drought, salinity, low temperatures, and others (Hamade et al., 2024; Li et al., 2025; Di Sario et al., 2025; Bhupenchandra et al., 2022). With broad applicability across various crops and horticultural plants, biostimulants can be applied via foliar spraying, soil drenching, or seed treatment to enhance nutrient uptake, stress tolerance, and overall plant vigor. Most biostimulants derive from natural sources, offering safety and environmental compatibility, which could effectively reduce chemical pesticide and fertilizer usage, mitigating pollution in soil, water systems, and other ecological environments. It aligns with the principles of green and sustainable agriculture, playing an indispensable role in ecological conservation and driving the green transformation of agricultural practices (Jamwal et al., 2025; Selim et al., 2025; Zaghloul et al., 2024; Asif et al., 2023; Fusco et al., 2022). However, traditional biostimulants exhibit dose-dependent affiliation, and their efficacies are significantly influenced by soil type, climate conditions, crop varieties, and application methods (Sanjuán et al., 2023; Ansari et al., 2023; Park et al., 2021; Johnson et al., 2022).

γ-Polyglutamic acid (γ-PGA) is a traditional biostimulant—a poly-γ-peptide polymer formed by the linkage of glutamic acid monomers via γ-carboxyl groups (Park et al., 2021; Johnson et al., 2022). Predominantly found in microbial capsules, it can be produced through microbial fermentation (Mu et al., 2021; Xu et al., 2020; Ma et al., 2022). Owing to its unique molecular structure, it exhibits exceptional water-binding capacity and metal ion chelation properties, making it widely applicable for soil amendment, fertilizer slow-release, and enhancing plant stress resistance (Xu et al., 2016; Bai et al., 2020; Bai et al., 2022). Previous studies have demonstrated that γ-polyglutamic acid (γ-PGA) functions through dual mechanisms of molecular chelation and physiological regulation. The free carboxyl groups on its molecular chain effectively chelate soil nutrients (e.g., nitrogen, phosphorus, potassium) and heavy metal ions (e.g., Cd^2+^/Pb^2+^), while promoting the proliferation of arbuscular mycorrhizal fungi and improving the rhizosphere microenvironment to enhance nutrient uptake by crops. In saline soil remediation, γ-PGA mitigates salt stress by binding Ca^2+^/Mg^2+^ ions and activates the plant antioxidant defense system (e.g., reducing malondialdehyde content and increasing proline levels). Furthermore, co-application of γ-PGA with organic fertilizers synergistically improves water and nitrogen use efficiency, and its slow-release degradation characteristics help alleviate soil microbial toxicity. These functions highlight the broad application potential of γ-PGA in soil improvement, pollution remediation, and enhancing crop stress resistance (Li et al., 2023; Fu et al., 2024; Mu et al., 2021; Pang et al., 2018).

γ-Polyglutamic acid (γ-PGA) functions as a high-efficiency biostimulant. Through γ-carboxyl-mediated molecular chelation and slow-release properties, it synergistically enhances soil fertility by increasing rhizospheric total nitrogen, total phosphorus, and available potassium content, remediates contamination, and optimizes nutrient utilization. Simultaneously, it activates plant antioxidant systems, thereby promoting biomass accumulation. However, crude γ-PGA faces practical limitations including low absorption efficiency, poor stability, and weak rainfastness. This is attributed to: The large molecular size hindering penetration through the plant cuticle, limiting entry to intercellular spaces via passive diffusion; High-viscosity solutions prone to gel formation, causing uneven distribution after foliar application; Electrostatic repulsion between unmodified crude γ-polyglutamic acid and negatively charged leaf surfaces, increasing susceptibility to rain washing (Luo et al., 2016; Ogunleye et al., 2015).

Building on this foundation, the strategic application of biostimulants and nanomaterials has emerged as a promising frontier for enhancing crop resilience and productivity under challenging environmental conditions. As sustainable agriculture demands innovative solutions to combat abiotic stresses such as drought, salinity, and heavy metal contamination, nano-engineered biostimulants offer unique advantages (Khundi et al., 2025; Mahapatra et al., 2022). They function as potent bio-effectors that can modulate plant physiological processes, enhance nutrient use efficiency, and activate stress-responsive pathways. For instance, recent studies on zaxinone mimics (MiZax) have demonstrated their efficacy in promoting plant growth and stress adaptation under desert climate conditions, highlighting the potential of molecular design in creating next-generation biostimulants (Wang et al., 2022, 2023). Similarly, nano-formulations can serve as smart delivery systems that improve the bioavailability and targeted translocation of active compounds, thereby maximizing their beneficial effects while minimizing environmental losses. This synergistic integration of biotechnology and nanotechnology opens new avenues for developing precision bio-stimulation strategies tailored to address the pressing challenges of modern agriculture.

**Scheme 1:**
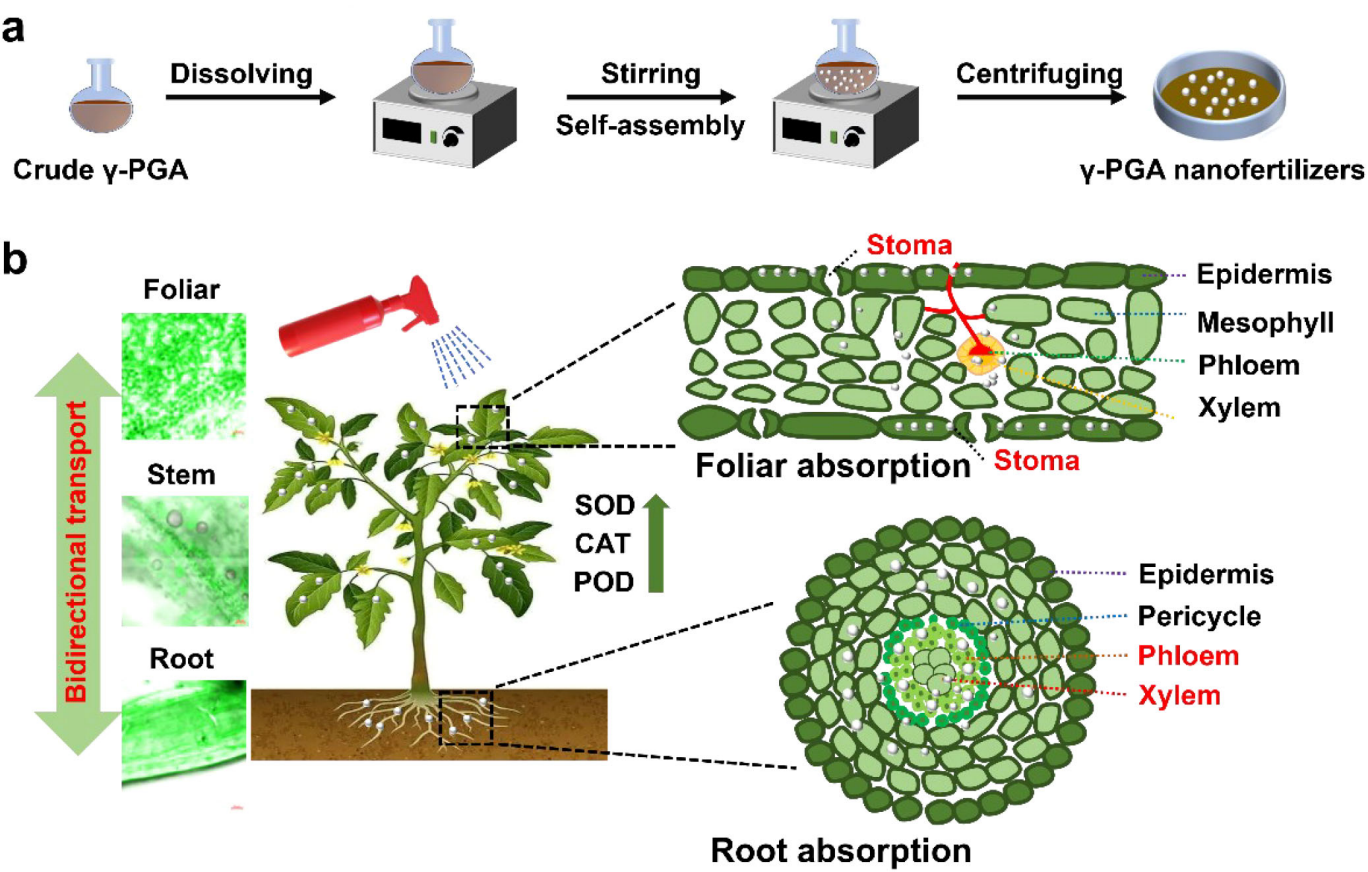
**(a)** Illustration for the synthesis of PGA nanofertilizers, **(b)** bidirectional conduction of PGA nanofertilizers in tomato plants.

This study aims to address the low utilization efficiency by converting crude γ-PGA into PGA nanofertilizers via one-step self-assembly technology (**Scheme 1a**). We hypothesize that this nano-engineering approach will optimize the particle size and morphology of γ-PGA, thereby enhancing its foliar retention, uptake efficiency, and systemic mobility within plants, ultimately leading to improved utilization efficacy for tomato plant growth (**Scheme 1b**), which will offer a novel pathway for developing high-performance and eco-friendly biostimulants.

## 2 Experimental sections

### 2.1 Synthesis of γ-polyglutamic acid nanofertilizers

Accurately 4000 mg of crude γ-polyglutamic acid (γ-PGA) powders were dissolved in 50 mL deionized water and magnetically stirred for 24 h. The solution was centrifuged at 10000 r/min (approximately 9,400 × g) for 15 min at room temperature, washed three times with distilled water, and vacuum-dried at room temperature to obtain PGA nanofertilizers.

### 2.2 Uptake and translocation of PGA nanofertilizers in tomato plants

FITC (fluorescein isothiocyanate, isomer I) labeling was performed by dissolving PGA nanofertilizers and FITC in deionized water at a mass ratio of 10:1, followed by magnetic stirring for 12 h at room temperature in the dark. The mixture was then centrifuged at 10,000 rpm for 15 min, washed three times with distilled water, and vacuum-dried at 60°C for 12 h in dark to obtain the FITC labeled PGA nanofertilizers.

Tomato seedlings were grown in a growth chamber under a 16/8 h light/dark cycle at 24°C and 60% relative humidity. Each experiment included three independent biological replicates. Uniform tomato seedlings at the 4–5 leaf stage were used. Tomato seedlings’ roots or leaves were separately immersed in 0.5 mg/mL FITC-labeled PGA nanofertilizers solutions with 0.1% tween-80 surfactant (deionized water with 0.1% tween-80 surfactant as control) at room temperature. After 24 h, roots, stems, and leaves were harvested. Root, stem, and leaf segments (1 cm in length) were hand-sectioned using a sharp razor blade and immediately mounted in deionized water to minimize artifacts from tissue damage. Prior to observation, all samples were gently rinsed with deionized water for 1 min to remove the PGA nanoparticles adhered on the surface of leaves, and fixed on a glass slide, then observed under confocal laser scanning microscopy to analyze PGA nanofertilizers absorption and translocation. The excitation wavelength of FITC was 488 nm. Each experiment was repeated three times.

### 2.3 Rainfastness washing evaluation of foliar-applied PGA nanofertilizers

Fresh tomato leaves were immersed in either 0.5 mg/mL FITC-labeled PGA nanofertilizers or free FITC aqueous solution with 0.1% tween-80 surfactant at identical concentrations. After air-drying, a shower device was used to simulate rainfall on leaves with consistent water volume per tomato seedling in a pot. Following secondary air-drying, leaf samples were examined via confocal microscopy to observe the retention of FITC and FITC@PGA nanofertilizers. The excitation wavelength of FITC was 488 nm. Each experiment was repeated three times.

The detention rate is calculated according to the following formula:


$$\begin{array}{c} \text{Retention rate }\%\\ =\frac{\text{Amount of after washed leaves}-\text{Initial amount of leaves}}{\text{Amount of before washed leaves}-\text{Initial amount of leaves}}\times 100 \end{array}$$

### 2.4 Foliar application on tomato seedlings

Uniform tomato seeds were surface-sterilized with 2% sodium hypochlorite for 10 min, followed by thorough rinsing. The sterilized seeds were placed on filter paper in Petri dishes and incubated in a growth chamber at 22°C/24°C with 60% humidity. After germination, seedlings were transplanted into nursery pots. Beginning at 14 days post-transplantation, plants were foliar-sprayed every 3 days with either 10, 20, and 30 μg/mL aqueous solution with 0.1% tween-80 surfactant of crude γ-PGA or PGA nanofertilizers respectively, alongside a deionized water as control group. Nine days after initial treatment, the following parameters were measured: plant height, stem diameter, leaf area, chlorophyll content, leaf thickness, total root length, root tip number, root surface area, root volume, and enzyme activities. All treatments were replicated three times.

### 2.5 Determination of growth phenotypes of tomato plants after foliar application with PGA nanofertilizers

Plant height was measured from base to apical meristem using a ruler; stem diameter, leaf area, chlorophyll content, and leaf thickness were determined with vernier calipers, a portable chlorophyll meter, and leaf thickness gauge, respectively; root parameters (total length, volume, surface area, tip number) were analyzed using a root scanner. All treatments were replicated three times.

### 2.6 Determination of biochemical indicators in tomato plants after foliar application of PGA nanofertilizers

#### 2.6.1 Determination of peroxidase activity

Leaf samples (0.10 g) from differentially treated tomato seedlings were homogenized in liquid nitrogen with 1 mL of extraction buffer (2-8°C). The homogenate was centrifuged (4°C×12000 r/min, 10 min), and the supernatant was collected and filtered by 0.45 μm filter membrane. POD activity was quantified using a Peroxidase Assay Kit (Griess Biotechnology Co., Ltd., Suzhou, China). Absorbance was measured at 470 nm using a microplate reader after termination of the color reaction. All assays were performed in triplicate.

#### 2.6.2 Determination of superoxide dismutase activity

Leaf tissues (0.10 g) from various treatment groups were pulverized in liquid nitrogen with 1 mL of cold extraction buffer (2-8°C). Following centrifugation (4°C×12000 r/min, 10 min)), the supernatant was filtered by 0.45 μm filter membrane and subjected to SOD activity analysis using a Superoxide Dismutase Detection Kit (Griess Biotechnology Co., Ltd.). Absorbance readings were taken at 450 nm with a microplate reader. Triplicate measurements were conducted.

#### 2.6.3 Determination of catalase activity

Tomato seedling leaves (0.10 g per sample) were cryogenically ground in 1 mL of extraction buffer (2-8°C) using liquid nitrogen. The homogenate was centrifuged ((4°C×12000 r/min, 10 min)), and the supernatant was filtered by 0.45 μm filter membrane and assayed for CAT activity with a Catalase Test Kit (Griess Biotechnology Co., Ltd.). Absorbance was recorded at 510 nm using a microplate reader. Three technical replicates were implemented.

#### 2.6.4 Determination of malondialdehyde content

Leaf samples (0.10 g) underwent homogenization in liquid nitrogen with 1 mL of chilled extraction buffer (2-8°C). After centrifugation (4°C×12000 r/min, 10 min), the supernatant was filtered by 0.45 μm filter membrane and analyzed for MDA content using a Malondialdehyde Assay Kit (Griess Biotechnology Co., Ltd.). A dual-wavelength measurement was performed at 532 nm and 600 nm with a microplate reader. Triplicate determinations were carried out.

### 2.7 Data analysis

All treatments were run with at least 3 replicates. Comparisons of the biochemical indicators were analyzed using one-way analysis of variance (ANOVA) and Duncan’s multiple range test. ^*^P < 0.05 and ^**^P < 0.01 were considered as significant and highly significant, respectively. The statistical analyses were performed using Data Processing Station SPSS 22.0 (IBM, USA). The charts and graphs of data were presented by OriginPro 8.5 (OriginLab, USA). Graphed data are shown as means ± standard errors.

## 3 Results and discussion

### 3.1 Synthesis and characterization of PGA nanofertilizers

**Figure 1:**
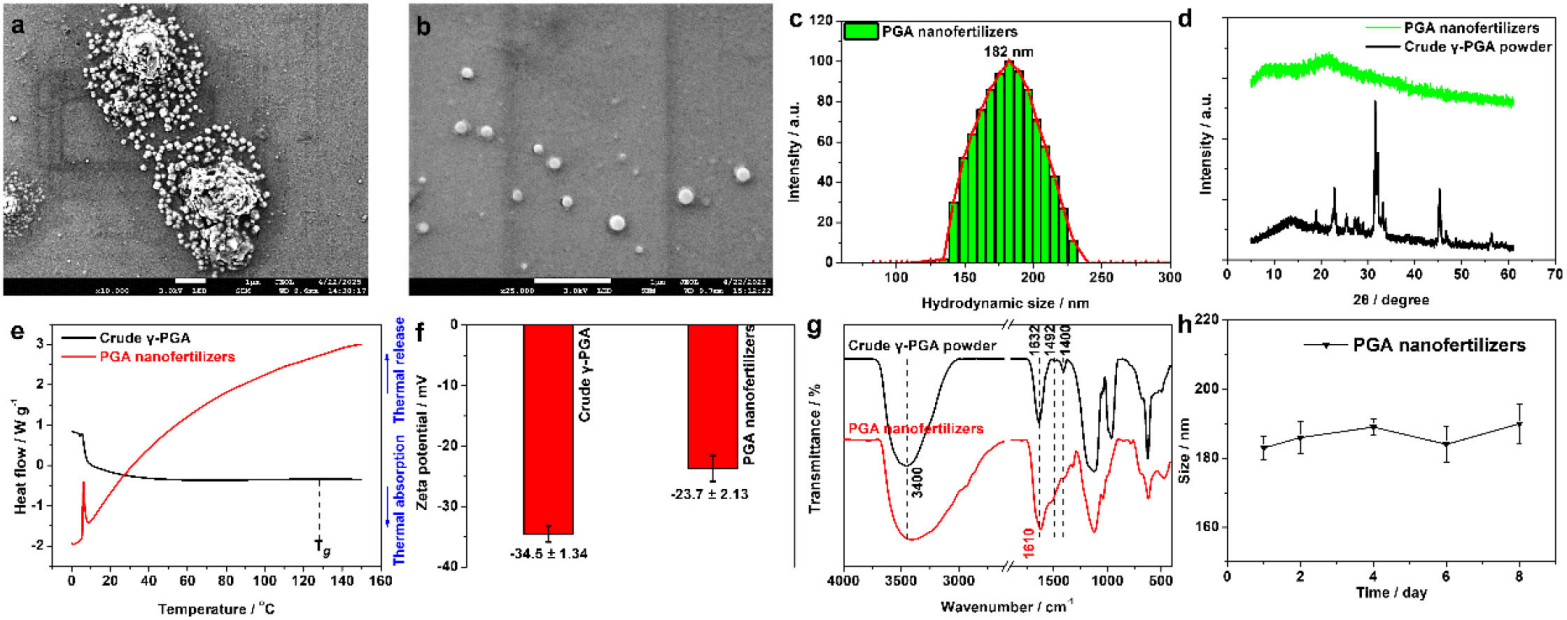
**(a, b)** SEM images of PGA nanofertilizers at different magnification scales, **(c)** DLS, **(d)** XRD, **(e)** DSC, **(f)** zeta potential, **(g)** FTIR figures of PGA nanofertilizers and crude γ-PGA powder; **(h)** hydrodynamic sizes of the prepared PGA nanofertilizers in aqueous solution on different days (*n* = 3).

Under deionized-water conditions without added salts, this study developed a facile one-step self-assembly strategy to yield uniform spherical PGA nanoparticles. Scanning electron microscopy (SEM) revealed that the PGA nanofertilizers exhibited uniform spherical morphology with an approximate diameter of 168 nm (**Figures 1a, b**). Dynamic light scattering (DLS) is used to measure the nanoparticle size distribution and stability in solutions or suspensions. Measuring the particle size distribution at different times, the trend of nanoparticles sedimentation over time can be demonstrated. To conduct the DLS test, 0.01 mg/mL PGA nanofertilizers were dispersed in the aqueous solution and filtered through 0.45 μm membrane. The PGA nanofertilizers exhibited a narrow particle size distribution with a hydrated particle size of 182 nm by DLS owing to the hydrogen bond interaction with water molecules (**Figure 1c**). The polydispersity index (PDI) is 0.143, less than 0.2, indicating that they appeared good dispersibility. Powder X-ray diffraction (XRD) patterns were performed on a Philips PANalytical X’pert diffractometer with a Cu-Kα radiation (λ = 0.15405 nm), which can precisely determine the crystal structure, texture and stress of the substance. Amorphous structures possess high-energy states (high free energy) and disordered molecular arrangements (weak intermolecular forces). During the dissolution process, the energy required to overcome the weak intermolecular forces is relatively low, which leads to loaded molecules can more readily dissociate from the bulk and enter the solution (Hejazi et al., 2023; Zhang et al., 2025). Additionally, the disordered structure provides a larger surface area and facilitates faster molecular diffusion rate (Ioelovich, 2021; Shen et al., 2018). XRD patterns (scan range: 5°-60°; scan speed: 2°/min; step width: 0.01°) indicate markedly reduced crystallinity consistent with a largely amorphous or highly disordered state under these conditions (**Figure 1d**). It was also verified through the DSC patterns (eliminate the thermal history at a rate of 10°C/min, then raise to 150°C/min; keep warm for 3–5 min; cool at a rate of 50°C/min until 0°C; measure at a rate of 10°C/min, and raise to 150°C.). DSC patterns shown that PGA nanofertilizers appeared no obvious strong thermal events, restricted molecular chain movement, crystallization being inhibited, and significant improvement in thermal stability compared with crude γ-PGA powder. The reason may be that 1) during the nanomization process, the hydrophilic groups of γ-PGA are occupied or encapsulated, resulting in a decrease in hygroscopicity; 2) the nanocomposite structure may restrict the movement of polymer molecular chains, thereby inhibiting the processes of cold crystallization; 3) the PGA nanofertilizers may have formed an amorphous state or stable complexes, making them insensitive to heat (**Figure 1e**). XRD and DSC patterns indicate a strong reduction in crystallinity consistent with a largely amorphous or highly disordered state. Suppressed XRD peaks together with inhibited crystallization in DSC are consistent with reduced crystallinity in polymer nanomaterials. However, without orthogonal structural methods (e.g., TEM/SAED, Raman, ssNMR), nanocrystallinity or size-induced peak broadening cannot be fully excluded. The zeta potential of crude γ-PGA powder in water was -34.5 mV owing to the ionization of a large number of free carboxyl groups (-COO^−^) on the molecular chain of γ-PGA (**Figure 1f**). While the absolute value of zeta potential for PGA nanofertilizers in water decreased from 34.5 mV to 23.7 mV (**Figure 1f**) which might be because the -COO^−^ of γ-PGA are occupied or encapsulated within the nanospheres. The broad bands at 3400 and 1632 cm^-1^ in the FTIR spectrum of crude γ-PGA powder were primarily ascribed to the stretching vibration of O-H/N-H and C=O amide I band, respectively (**Figure 1g**). The weak bands at 1492 and 1400 cm^-1^appeared, owing to the bending vibration of N-H and symmetrical stretching of carboxylate group in crude γ-PGA powder (**Figure 1g**). While the peak intensity has weakened at 1492 and 1400 cm-1 in that of PGA nanofertilizers, which also might be because the -COO^−^ of γ-PGA are occupied or encapsulated within the nanospheres. The size distribution and stability of nanoparticles are important indicators for the application of the prepared PGA nanofertilizers. Obviously, the PGA nanofertilizers remained well-dispersed for at least 8 days without aggregation and the size distribution could maintain unvaried as proved by DLS (**Figure 1h**). The PDI on different days are all less than 0.2 (**Supplementary Table S1**). Although, the uniformity is supported by SEM (**Figures 1a, b**) and DLS (182 nm, PDI 0.143) with 8-day stability under controlled (deionized water) conditions (**Figure 1h**), batch-to-batch yield and stability of PGA nanofertilizers under realistic environmental variations (e.g., ionic strength, pH, organic matter) were not systematically examined here and will be optimized in future work. Compared to the well-defined crystalline structure of crude γ-PGA powder, the largely amorphous or highly disordered state of PGA nanofertilizers is expected to exhibit higher solubility and dissolution rates, which are crucial for dispersion, stability, and uptake efficiency as active ingredients on crop leaf surfaces.

### 3.2 Uptake and translocation of polyglutamic acid nanofertilizers in tomato plants

**Figure 2:**
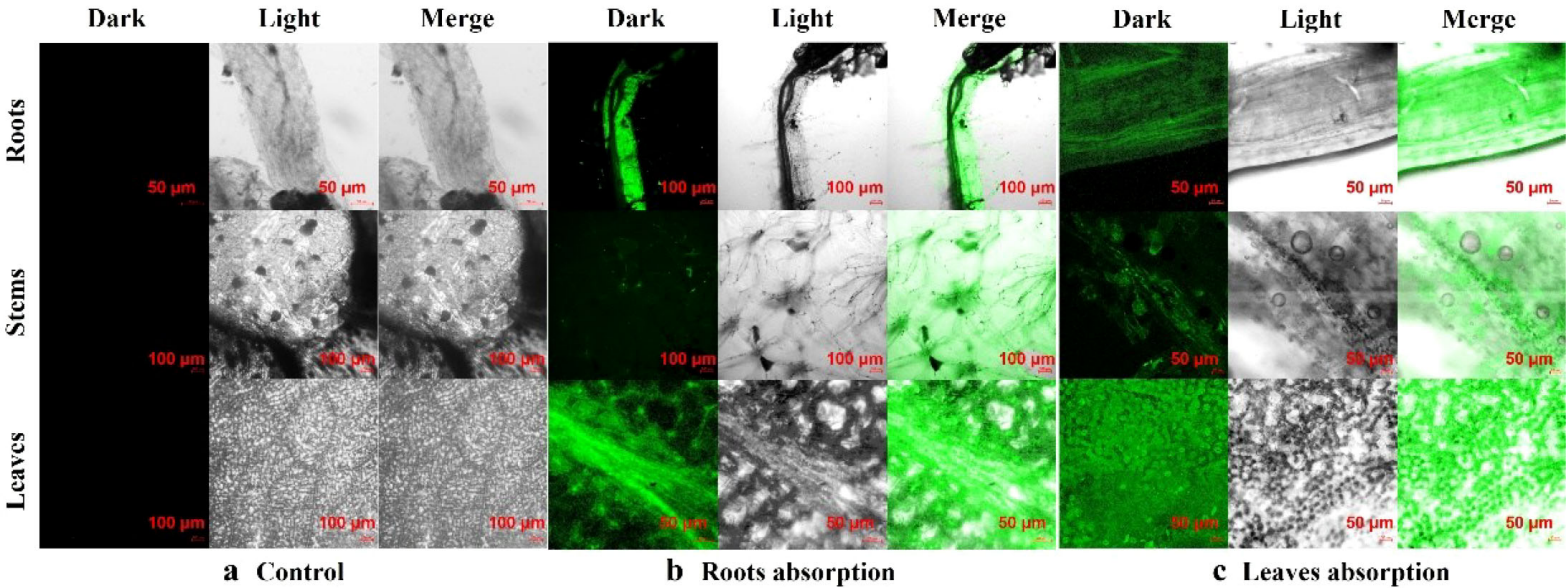
Confocal microscope images of absorption and transport of FITC labeled PGA nanofertilizers (Noted as FITC@PGA) in tomatoes (roots, stems, and leaves) in various parts of tomatoes: **(a)** control treatment group, **(b)** via root absorption, **(c)** via leaves absorption. The excitation wavelength of FITC was 488 nm. Each experiment was repeated three times.

Nanofertilizers show great potential for sustainable use in terms of soil fertility, crop production, and minimal or no environmental trade-offs. Due to their submicroscopic size, nanofertilizers have a large surface area-to-volume ratio, along with nutrient encapsulation capabilities and enhanced mobility, which enable them to improve plants’ nutrient uptake and increase crop yields (Jakhar et al., 2022; Chakraborty et al., 2023). This study employed FITC-labeled PGA nanofertilizers (FITC@PGA) to investigate their uptake and translocation in tomato plants. Roots or leaves of separate tomato plants were immersed in aqueous suspensions containing FITC@PGA nanofertilizers, with deionized water as the control (**Figure 2a**), and fluorescence distribution was observed using confocal laser scanning microscopy. Before observation, all samples were gently rinsed with deionized water for 1 min to remove the PGA nanoparticles adhered on the surface of leaves. For root-treated plants: Roots exhibited intense green fluorescence concentrated in epidermal and cortical cells, particularly near and within vascular tissues (**Figure 2b**), indicating PGA nanofertilizers attachment and absorption by root cells and entry into xylem vessels responsible for upward transport of water and nutrients. Stems showed fluorescence signals primarily localized in vascular bundles and xylem, demonstrating that root-absorbed PGA nanofertilizers were transported upward via transpiration-driven flow from roots to shoots (**Figure 2b**). Leaves displayed fluorescence signals concentrated in veins and surrounding mesophyll cells (**Figure 2b**), confirming that PGA nanofertilizers absorbed by roots were translocated over long distances through root xylem and stem vascular systems to apical leaf tissues. For foliar-treated plants: Leaves revealed strong fluorescence signals penetrating the cuticle, epidermis, palisade, and spongy mesophyll, with accumulation near vascular bundles (**Figure 2c**), indicating PGA nanofertilizers penetration through leaf surface barriers into internal tissues. Stems exhibited fluorescence signals in phloem and throughout vascular bundles (**Figure 2c**), demonstrating that leaf-absorbed PGA nanofertilizers utilized phloem for systemic movement. Roots displayed distinct green fluorescence (**Figure 2c**), proving that foliar-absorbed PGA nanofertilizers underwent long-distance translocation to root systems. Fluorescence imaging of FITC@PGA indicates systemic, vascular-localized signals consistent with bidirectional movement from absorption of both roots and leaves. As previously reported in the literature (Liang et al., 2021; Liu et al, 2023), the distribution of free FITC was more diffuse, and there was no specific localization to the vascular system. While during uptake and transport, partial FITC released from FITC@PGA cannot be excluded. therefore, fluorescence may reflect both nanoparticle-associated and free dye signals, warranting further verification. Fluorescence imaging demonstrated vascular-localized signals; attribution to intact nanoparticles requires additional verification.

### 3.3 Enhanced rainfastness washing performance of PGA nanofertilizers

**Figure 3:**
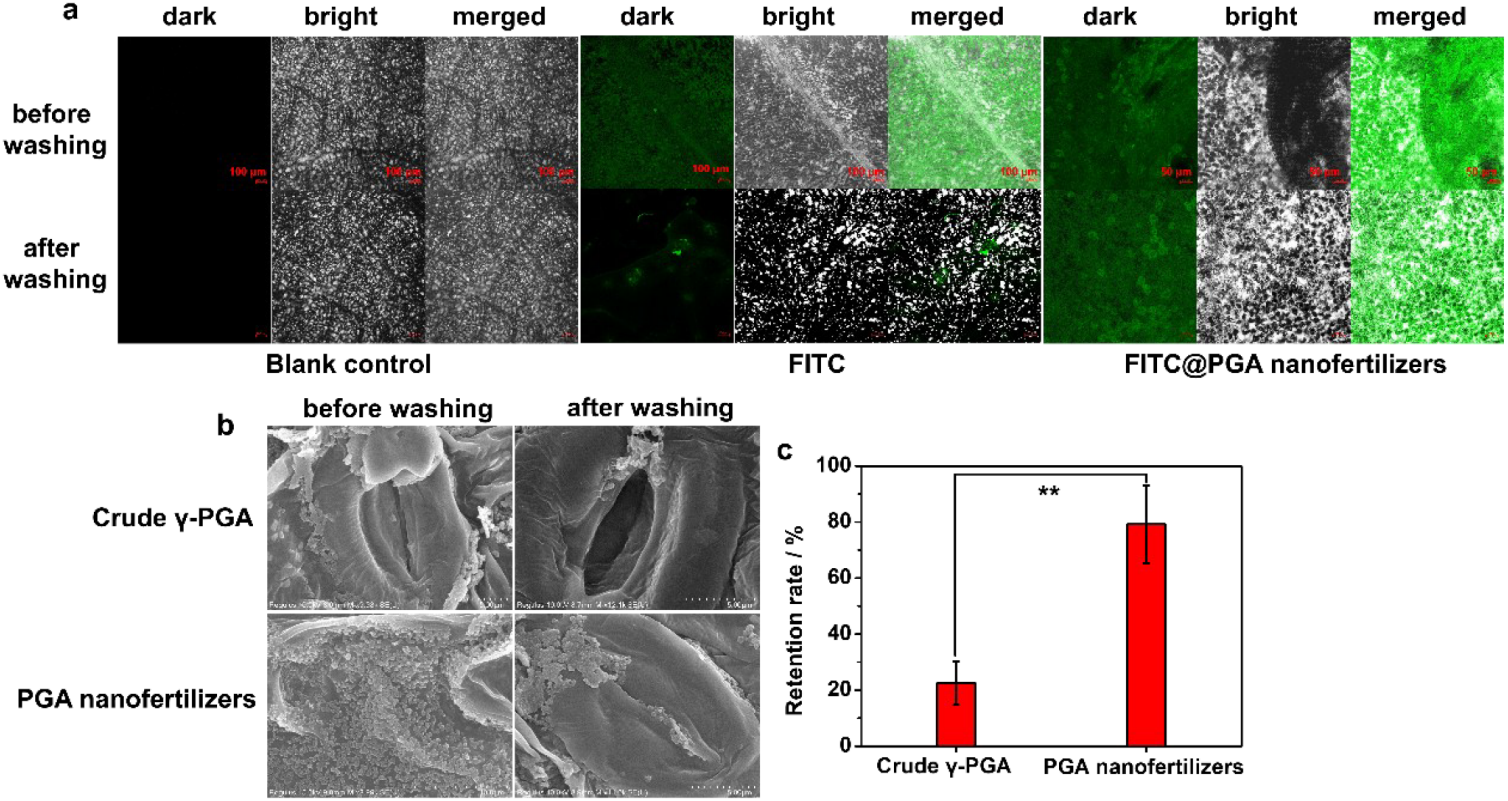
**(a)** Laser confocal imaging of free FITC and FITC@PGA nanofertilizers, **(b)** SEM photographs of crude γ-PGA and PGA nanofertilizers, **(c)** the retention rate of γ-PGA and PGA nanofertilizers on tomato leaf surface before and after washing with water, respectively. Each experiment was performed three times (*n* = 3). The data are the mean values ± standard errors. Comparisons of the retention rate results were analyzed using one-way analysis of variance (ANOVA) and Duncan’s multiple range test. One star in graphs means that the comparison is significant at *P* < 0.05, and two stars means that the comparison is significant at *P* < 0.01. The excitation wavelength of FITC was 488 nm.

Traditional fertilizers are prone to loss due to rainwater, primarily manifested in the easy leaching of their water-soluble nutrients. When encountering rainfall or irrigation, the soluble nutrients in the fertilizer rapidly dissolve in the water. A portion of these dissolved nutrients are washed away by surface runoff, while another portion leaches down with the water (leaching) into deeper soil layers beyond the reach of crop roots. It not only causes significant nutrient loss, reducing fertilizer utilization efficiency and increasing agricultural production costs, but also may lead to water pollution (Liu et al., 2024; Comber et al., 2025; Niu et al., 2024; Yang et al., 2025). This paper evaluated the rainfastness washing and leaf adhesion by comparing the retention of free FITC and crude γ-PGA versus FITC-labeled PGA nanofertilizers and PGA nanofertilizers on tomato leaves before and after simulated rainfall (leaves at a 45° bevel to the horizontal; total spraying time: 3 min; height from the leaf surface: 1.0 m; initial velocity: 0 m/s; mimicked rainfall intensity: 3 mm/h). Confocal laser scanning microscopy (**Figure 3a**) revealed significantly stronger retention (fluorescence intensity 73.54%) of FITC@PGA nanofertilizers than that (fluorescence intensity 9.78%) of free FITC on leaf surfaces after rain wash-off, which is 7.52 times compared with fluorescence intensity of free FITC. Further, the retention ability of PGA nanofertilizers on tomato leaf surfaces was observed by SEM before and after washing. It could be seen that there was still a large amount of PGA nanofertilizers retained on the leaf surface (**Figure 3b**, down). The retention rate of PGA nanofertilizers after washing was 3.52 times higher than that of crude γ-PGA, which was shown a highly significant difference (^**^P < 0.01) (**Figure 3c**). The high post-rain retention of PGA nanofertilizers stemmed from efficient foliar absorption—their nanoscale size enabled facile penetration into leaves through cuticular pores, microcracks in the wax layer, and epidermal cell junctions. As a polymeric biomaterial, PGA nanofertilizers formed continuous films upon dispersion on leaves via chain entanglement, diffusion, and solvent evaporation. These films exhibited viscoelasticity and flexibility, tightly encapsulating the leaf surface to create a protective layer that minimized rain-induced removal of active ingredients. Compared to crude γ-PGA, PGA nanofertilizers demonstrated enhanced dispersibility and more uniform film-forming capability. Crude γ-PGA, a high-molecular-weight water-soluble polymer, is primarily root-applied as a soil conditioner and nutrient enhancer. Its efficacy heavily depends on soil conditions and root absorption capacity, diminishing under poor root development or adverse soil environments. For leaf physiological stresses induced by drought, intense light, or pests/diseases, root-applied crude γ-PGA powder exerts only indirect and delayed mitigation effects. The exceptional rainfastness washing and leaf retention of PGA nanofertilizers significantly enhance the efficacy of γ-PGA foliar application, which will provide a novel approach for efficient and eco-friendly γ-PGA utilization in agriculture.

### 3.4 Effects of foliar application of PGA nanofertilizers for tomato plant growth

**Figure 4:**
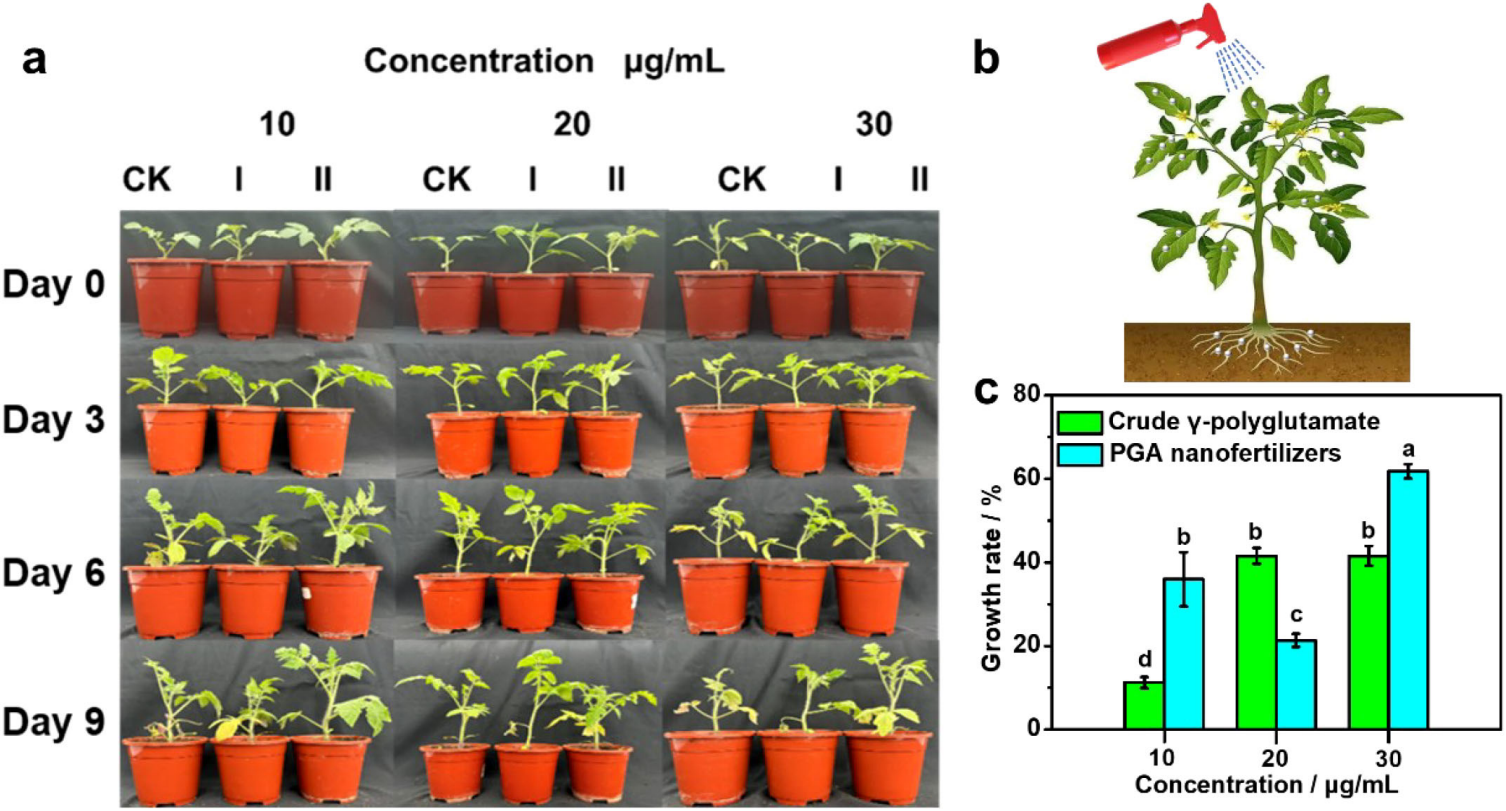
**(a)** Phenotypic photographs of tomato plants on day 9 after foliar application with crude γ-PGA (I) and PGA nanofertilizers (II), **(b)** schematic illustration of the foliar application process for PGA nanofertilizers, **(c)** plant height increase rate of tomatoes treated with different concentrations of PGA nanofertilizers compared with that of crude γ-PGA. Data are presented as means ± standard errors (*n* = 3). Comparisons of the control efficacy were analyzed using one-way analysis of variance (ANOVA) and Duncan’s multiple range test. Values marked with lowercases correspond to significant differences (*P* = 0.05) according to a least significant difference test.

This study investigated the dynamic effects of foliar-applied PGA nanofertilizers versus crude γ-PGA at varying concentrations (10, 20, and 30 μg/mL) on tomato growth phenotype (**Figure 4b**). Compared to control (CK) and crude γ-PGA treatments, plants treated with PGA nanofertilizers exhibited superior leaf glossiness and canopy architecture (**Figure 4a**). At 30 μg/mL, leaves treated with PGA nanofertilizers demonstrated significantly enhanced glossiness on day 9 compared with that of both CK and crude γ-PGA groups, which could be attributed to the exceptional leaf adhesion and retention of PGA nanofertilizers to improve the absorption efficiency of plants. Analysis of growth rates further demonstrated that PGA nanofertilizers significantly enhanced leaf expansion. Correspondingly, quantitative data on plant height increase rates (**Figure 4c**; **Supplementary Table S2**) indicated that the 30 μg/mL PGA nanofertilizers treatment resulted in a 61.80 ± 1.71% increase in plant height, representing a statistically significant enhancement compared with that of the crude γ-PGA group (41.57 ± 2.38%), which means that the PGA nanofertilizers can effectively promote cell elongation and division. Moreover, at equivalent concentrations, crude γ-PGA-treated leaves developed marginal chlorosis by day 9, whereas leaves treated with PGA nanofertilizers maintained structural integrity and healthier appearance, which presumably linked to the PGA nanofertilizers-enhanced antioxidant enzyme activities.

### 3.5 Effects of foliar application of PGA nanofertilizers on physiological and morphological indicators of tomato plants

The chlorophyll content is a core indicator that can reflect the photosynthetic capacity of plants, directly affecting the efficiency of organic matter synthesis. Biostimulants can enhance the photosynthetic rate by promoting chlorophyll synthesis by providing an energy basis for plant growth. Parameters such as plant height, stem diameter, and leaf area intuitively reflect the vegetative growth status of plants. The increase in plant height and stem diameter reflects the activity level of cell elongation and division, while the expansion of leaf area increases the light capturing area, further enhancing photosynthesis. The optimization of these indicators is an important sign of the healthy growth of plants. The root system morphology (including root volume, root surface area, number of root tips, etc.) determines the plant’s ability to absorb water and nutrients. A well-developed root system can more efficiently acquire soil resources and enhance the plant’s tolerance to stress. Leaf thickness is related to the differentiation and arrangement of mesophyll cells. Thicker leaves usually have more developed palisade and spongy tissues, which is beneficial for improving the light energy utilization efficiency and water retention capacity, and is an important morphological feature for plants to adapt to environmental changes (Khangura et al., 2020; Croft et al., 2017; Luo et al., 2019; Li et al., 2023).

**Figure 5:**
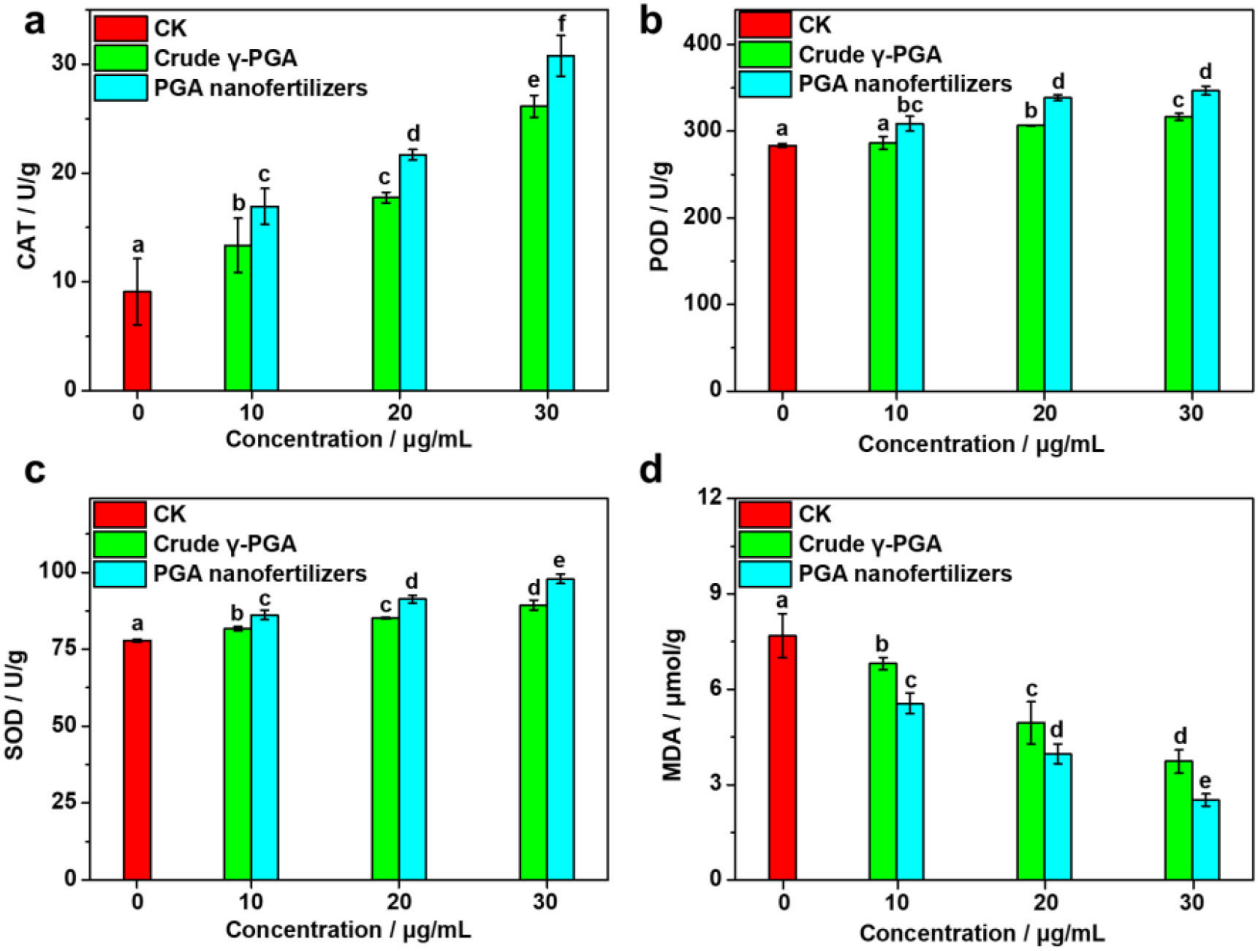
Biochemical indices in tomato leaves after foliar application with different concentrations of PGA nanofertilizers, crude γ-PGA, and control (CK): **(a)** catalase (CAT) activity, **(b)** peroxidase (POD) activity, **(c)** superoxide dismutase (SOD) activity, **(d)** malondialdehyde (MDA) content. Data are presented as means ± standard errors (*n* = 3). Comparisons of the physiological and biochemical levels were analyzed using one-way analysis of variance (ANOVA) and Duncan’s multiple range test. Values marked with lowercases correspond to significant differences (*P* = 0.05) according to a least significant difference test.

**Figure 6:**
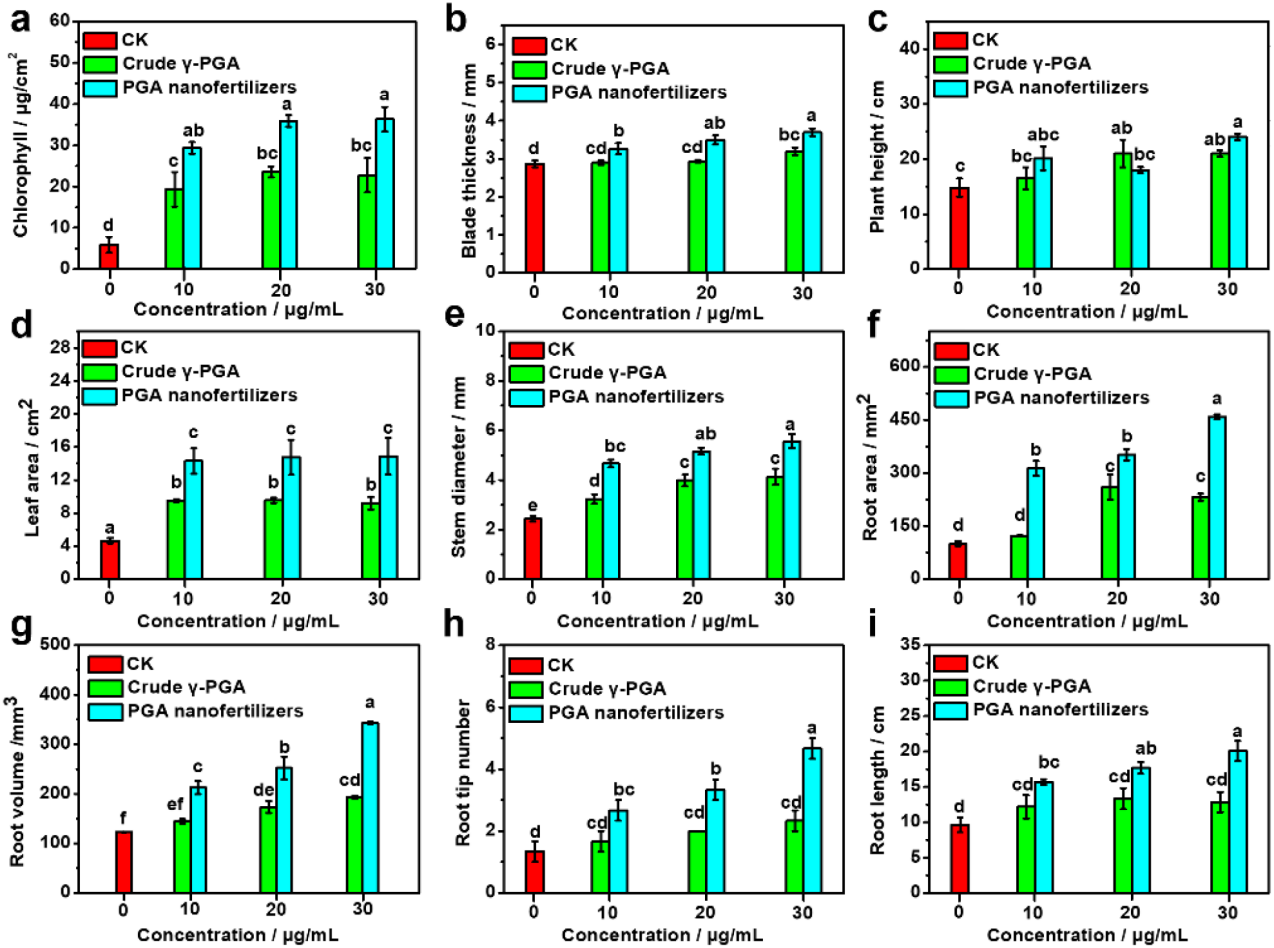
Physiological and morphological indicators of tomato plants after foliar application with different concentrations of PGA nanofertilizers, crude γ-PGA, and control (CK): **(a)** chlorophyll content, **(b)** leaf thickness, **(c)** plant height, **(d)** leaf area, **(e)** stem diameter, **(f)** root surface area, **(g)** root volume, **(h)** number of root tips, **(i)** root length. Data are presented as means ± standard errors (*n* = 3). Graphed data are shown as means ± standard errors (*n* = 3). Comparisons of the physiological and biochemical levels were analyzed using one-way analysis of variance (ANOVA) and Duncan’s multiple range test. Values marked with lowercases correspond to significant differences (*P* = 0.05) according to a least significant difference test.

This study evaluated physiological and morphological parameters in tomato plants based on foliar application of PGA nanofertilizers versus crude γ-PGA at concentrations (10, 20, and 30 μg/mL) (**Figure 5**; **Supplementary Table S3**). Compared to control (CK) and crude γ-PGA treatments, PGA nanofertilizers significantly enhanced physiological and morphological traits. For chlorophyll content (**Figure 6a**): PGA nanofertilizers-treated groups exhibited dose-dependent increases, peaking at 36.30 ± 2.99 μg/cm² (30 μg/mL)–representing a 515% increase over CK (5.90 ± 1.91 μg/cm²) and 60% over crude γ-PGA (22.77 ± 4.17 μg/cm²) at equivalent concentration. This enhancement is attributed to efficient PGA nanofertilizers absorption and translocation to mesophyll cells, where their nanoscale structure facilitates epidermal penetration, promoting photosynthetic pigment synthesis. While the data indicate a promising increase in chlorophyll meter readings following nanofertilizer treatment (**Figure 6a**), these measurements must be interpreted with caution. Portable chlorophyll meters provide a useful proxy but can be influenced by leaf surface properties. The presence of a nanofertilizer-derived surface film could potentially contribute to the observed higher values. Therefore, extraction-based assays will be required in subsequent research to confirm absolute pigment concentrations and validate this observed trend. For leaf area and blade thickness (**Figures 6b, d**): The 30 μg/mL PGA nanofertilizers group achieved maximum values (blade thickness: 3.70 ± 0.10 mm ; leaf area: 14.86 ± 2.24cm2), exceeding CK (blade thickness: 2.87 ± 0.09 mm, leaf area: 4.64 ± 0.33 cm2) by 320% and 129%, respectively. For plant height and stem diameter (**Figures 6c, e**): The 30 μg/mL PGA nanofertilizers group achieved maximum values (height: 24.00 ± 0.58 cm; diameter: 5.57±0.29 mm), exceeding CK (height: 14.83 ± 1.64 cm, diameter: 2.43 ± 0.12 mm) by 62% and 129%, respectively. Most notably, root development parameters showed remarkable improvement, with the 30 μg/mL nanofertilizer treatment yielding root area of 458.71 ± 6.70 mm^2^ (4.57 × CK, 1.98 × crude γ-PGA) (**Figure 6f**), root volume of 343.08 ± 3.20 mm^3^ (2.8 × CK, 1.8 × crude γ-PGA) (**Figure 6g**) and root tip number of 4.67 ± 0.33 (3.5 × CK, 2.0 × crude γ-PGA). These growth-promoting effects are closely associated with the unique absorption and translocation characteristics of the PGA nanofertilizers. Their nanoscale size (182 nm) facilitates transmembrane transport via apoplastic and symplastic pathways compared to crude γ-PGA. Importantly, the foliar-absorbed PGA nanofertilizers are translocated downward to the roots through the phloem, directly stimulating root apical meristem activity (**Figures 6h, i**). Furthermore, the PGA nanofertilizers may function as signaling molecules, activating the synthesis and distribution of hormones (such as auxins and cytokinins) through their bidirectional translocation. The significant increase in root tip number (**Figure 6h**) is likely related to the hormone balance regulation (e.g., elevated cytokinin levels) mediated by the downward transport of PGA nanofertilizers.

### 3.6 Effects of foliar application of PGA nanofertilizers on the stress resistance for tomato plants

Excess reactive oxygen species (ROS) accumulated in plants under stress conditions can trigger membrane lipid peroxidation and cellular damage. However, the antioxidant defense system, which is synergistically composed of superoxide dismutase (SOD), peroxidase (POD), and catalase (CAT), can efficiently quenched ROS through a cascade scavenging mechanism (SOD catalyzes O_2_^−^·→H_2_O_2_, and CAT/POD decomposes H_2_O_2_→H_2_O). This significantly inhibits the production of toxic products such as malondialdehyde (MDA) (a reduction of up to 67.2%), thereby protecting the integrity of cell membranes (Zhong et al., 2021; Blokhina et al., 2003; Gill and Tuteja, 2010; Wang et al., 2024].

Foliar application of PGA nanofertilizers could significantly enhance the antioxidant defense system in tomato leaves and mitigate membrane lipid peroxidation damage. Compared to the control and crude γ-PGA treatments, the nanofertilizers substantially elevated activities of key antioxidant enzymes in a concentration-dependent manner. At 30 μg/mL concentration, CAT activity reached 30.79 ± 1.86 U/g (17.7% higher than crude γ-PGA, 239% above control), while POD and SOD activities peaked at 347.00 ± 5.30 U/g and 97.91 ± 1.51 U/g, respectively (**Figures 5a-c**; **Supplementary Table S4**). Concurrently, malondialdehyde content decreased progressively with increasing nanofertilizer concentration, with 30 μg/mL group recording only 2.52 ± 0.19 μmol/g (a 67.2% reduction below control), confirming effective suppression of membrane peroxidation (**Figure 5d**; **Supplementary Table S4**). These protective effects are intrinsically linked to the bidirectional translocation of the PGA nanofertilizers: root-absorbed nanoparticles move upward to leaves via the xylem, while foliar-absorbed ones translocate downward to roots via the phloem, establishing a systemic antioxidant network. Their nanoscale size permits penetration across cellular and organellar membranes (e.g., chloroplasts, mitochondria), enabling targeted localization at primary ROS generation sites. This spatial advantage activates SOD-mediated disproportionation of superoxide radicals and supplies H_2_O_2_ substrate for the CAT/POD scavenging cascades. The PGA nanofertilizers may synergistically amplify the entire antioxidant enzyme system at relatively low concentrations, potentially through direct protein interactions or modulation of gene expression.

However, this study has several limitations. The experimental duration was relatively short, preventing assessment of long-term effects on tomato fruit yield; all experiments were conducted under controlled greenhouse conditions, and thus performance in complex and variable field environments requires further validation. Moreover, although γ-PGA is biodegradable, the long-term residue of nanoparticles in plant tissues and soil, along with their ecological impact, warrants more extensive investigation in future studies.

## 4 Conclusion

In summary, to enhance the efficacy of crude γ-PGA in field application, we developed PGA nanofertilizers via a one-step self-assembly strategy for the improvement tomato growth and stress resistance, which can transform the traditional root application of crude γ-PGA into an efficient and simplified foliar spraying. PGA nanofertilizers not only improve the solubility and dispersion stability of crude γ-PGA but also exhibit excellent rainfastness and leaf retention compared to crude γ-PGA. The prepared PGA nanofertilizers can be efficiently absorbed by tomato plants through both roots and leaves, with bidirectional translocation via xylem and phloem, thereby enhancing physiological activity. Physiological indices shown that foliar application of PGA nanofertilizers significantly enhanced chlorophyll content, root development, and antioxidant enzyme activities compared to that of crude γ-PGA, which led to significant improvement for tomato growth and stress tolerance. This simple, green nanoengineering strategy will provide a promising approach for sustainable biostimulant development and foliar fertilization.

## Data Availability

The datasets presented in this study can be found in online repositories. The names of the repository/repositories and accession number(s) can be found in the article/**Supplementary Material**.
