# Supplementary material for: Nanoengineered polyglutamic acid fertilizers via self-assembly for efficient tomato growth
Source: Front Plant Sci. 2026 Jan 15;16:1702462. doi: 10.3389/fpls.2025.1702462 (PMC12854139; doi:10.3389/fpls.2025.1702462)
Supplement: Supplementary file 1 [file Table1.docx]

**Nanoengineered Polyglutamic Acid Fertilizers via Self-Assembly for Efficient Tomato Growth**

Jiangtao Dong,^a,b*^ Hexin Li,^b^ Bowen Yuan,^b^ Donghui Zhang,^b^ Hongliang Wang,^b^ Tao Wang,^c^ Songwei Li,^b*^ Runqiang Liu,^a,b*^

^a^ *Henan Engineering Research Center of Green Pesticide Creation & Intelligent Pesticide Residue Sensor Detection, Henan Institute of Science and Technology, Xinxiang, 453003, Henan Province, People’s Republic of China.*

^b^ *School of Plant Protection and Environment,* *Henan Institute of Science and* ^b^ *Technology, Xinxiang, 453003, Henan Province, People’s Republic of China.*

^c^ *School of Chemistry and Chemical Engineering*, *Henan Institute of Science and Technology, Xinxiang, 453003, Henan Province, People’s Republic of China.*

^*^Corresponding authors:

*E-mail addresses*: 869644962@qq.com (J. Dong), [lear9999@163.com](mailto:lear9999@163.com) (S. Li), [liurunqiang1983@126.com](mailto:liurunqiang1983@126.com) (R. Liu).

**Table S1.** Hydrodynamic sizes of the prepared PGA nanofertilizers in aqueous solution on different days (*n* = 3).

| day | hydrodynamic size [nm] | PDI |
| --- | --- | --- |
| 1 | 183 ± 3.4 | 0.118 |
| 2 | 186 ± 4.7 | 0.127 |
| 4 | 189 ± 2.4 | 0.086 |
| 6 | 184 ± 5.1 | 0.134 |
| 8 | 190 ± 5.6 | 0.149 |

**Table S2**. The increase rate of plant height compared with the control after foliar spraying of PGA nanofertilizers and crude γ-PGA at different concentrations on tomato leaves at day 9 (*n* = 3).

| Sample | Concentrations  (μg/mL) | Plant height increase rate (%) |
| --- | --- | --- |
| CK | - | - |
| Crude γ-PGA | 10 | 11.23 ± 1.37 |
|  | 20 | 41.57 ± 1.92 |
|  | 30 | 41.57 ± 2.38 |
| PGA nanofertilizers | 10 | 35.96 ± 6.49 |
|  | 20 | 21.35 ± 1.55 |
|  | 30 | 61.80 ± 1.71 |

Note：The values in the table represent the mean ± standard error. According to ANOVA Tukey test, values labeled with lowercase letters correspond to significant differences (P = 0.05) according to a least significant difference test.

**Table S3**. The physiological and morphological indices of PGA nanofertilizers (NPs) with different concentrations sprayed on the surface of tomato leaves compared with that of the corresponding crude γ-PGA and CK (*n* = 3).

| Sample | Concentrations(μg/mL) | chlorophyll content  (μg/cm^2^) | plant height  (cm) | blade thickness  (mm) | leaf area  (cm^2^) | stem diameter  (mm) | root area  (mm^2^) | root volume  (mm^3^) | root tips numbe | root length  (cm) |
| --- | --- | --- | --- | --- | --- | --- | --- | --- | --- | --- |
| CK | - | 5.90 ± 1.91d | 14.83 ± 1.64c | 2.87 ± 0.09d | 4.64 ± 0.33a | 2.43 ± 0.12e | 100.30 ±6.66d | 123.47 ±3.30f | 1.33 ± 0.33d | 9.63 ± 1.04d |
| Crude γ-PGA | 10 | 19.33 ± 4.17c | 16.50 ± 2.02bc | 2.90 ± 0.06cd | 9.51 ± 0.199 | 3.23 ± 0.19d | 122.17 ± 1.47d | 144.24 ± 4.82ef | 1.67 ± 0.33cd | 12.18 ± 1.65cd |
|  | 20 | 23.53 ± 1.30bc | 20.17 ± 2.17abc | 2.93±0.03cd | 9.53 ± 0.33b | 3.98 ± 0.24c | 259.98 ± 35.41c | 173.54 ± 12.23de | 2.00 ± 0.00cd | 13.33 ± 1.50cd |
|  | 30 | 22.77 ± 4.17bc | 21.00 ± 0.58ab | 3.20 ± 0.10bc | 9.19 ± 0.77b | 4.13 ± 0.32c | 231.66 ± 9.47c | 193.06 ± 3.12cd | 2.33 ± 0.33cd | 12.84 ± 1.42cd |
| PGA nanofertilizers | 10 | 29.37 ± 1.41ab | 18.00 ± 0.58bc | 3.27 ± 0.15b | 14.30 ± 1.54c | 4.67 ± 0.16bc | 313.12 ± 21.11b | 212.71 ± 13.39c | 2.67 ± 0.33bc | 15.69 ± 0.40bc |
|  | 20 | 35.90 ± 1.41a | 21.00 ± 2.52ab | 3.50 ± 0.127ab | 14.74 ± 2.122c | 5.17 ± 0.14ab | 351.37 ± 15.78b | 252.26 ± 22.97b | 3.33 ± 0.33b | 17.69 ± 0.85ab |
|  | 30 | 36.30 ± 2.99 a | 24.00 ± 0.58a | 3.70 ± 0.10a | 14.86 ± 2.24c | 5.57 ± 0.29a | 458.71 ± 6.70a | 343.08 ± 3.20a | 4.67 ± 0.33a | 20.08 ± 1.42a |

Note：The values in the table represent the mean ± standard error. According to ANOVA Tukey test, values labeled with lowercase letters correspond to significant differences (P = 0.05) according to a least significant difference test.

**Table S4**. The biochemical indices of PGA nanofertilizers with different concentrations sprayed on the surface of tomato leaves compared with that of the corresponding crude γ-PGA and CK (*n* = 3)

| Sample | Concentrations  (μg/mL) | CAT  (U/g) | POD  (U/g) | SOD  (U/g) | MDA  (μmol/g) |
| --- | --- | --- | --- | --- | --- |
| CK | - | 9.08 ± 3.05a | 283.67 ± 2.31a | 77.85 ± 0.50a | 7.69 ± 0.69a |
| Crude γ-PGA | 10 | 13.37 ± 2.50b | 286.33 ± 7.50a | 81.66 ± 0.76b | 6.81 ± 0.19b |
|  | 20 | 17.75 ± 0.48c | 306.67 ± 0.58b | 85.15 ± 0.30c | 4.95 ± 0.66c |
|  | 30 | 26.15 ± 1.02e | 316.67 ± 4.16c | 89.44 ± 1.60d | 3.73 ± 0.37d |
| PGA nanofertilizers | 10 | 16.94 ± 1.64c | 308.67 ± 8.62bc | 86.14 ± 1.54c | 5.56 ± 0.32c |
|  | 20 | 21.68 ± 0.48d | 338.67 ± 3.51d | 91.30 ± 1.32d | 3.97 ± 0.31d |
|  | 30 | 30.79 ± 1.86f | 347.00 ± 5.30d | 97.91 ± 1.51e | 2.52 ± 0.19e |

Note：The values in the table represent the mean ± standard error. According to ANOVA Tukey test, values labeled with lowercase letters correspond to significant differences (P = 0.05) according to a least significant difference test.
